# 

**Published:** 1900-05-12

**Authors:** 


					iuc slaiiu hra ~oi aigdesi' "p urity." ?uccizc&t. ottay j^nr, i?uor
CADBURY'S if HI* OAD?y?YS
r.nnrtA >?- ^ v -
cocoa m> ^ cocoa
ABSOLUTELY PUSS. c~~~^ NO DRUGS OR ALKALIES USED.
No. 711. Vol. XXVIII. [??SSJ?] Saturday,
May 12, 1900.
[Subscription Terns,J gK,ICI|^WOP-EJSrC EL

				

